# Antineutrophil Cytoplasmic Autoantibodies Specific to Bactericidal/Permeability-Increasing Protein: A Cross-Road Between Prolonged Gram-Negative Bacterial Infections and Ulcerative Colitis/Primary Sclerosing Cholangitis

**DOI:** 10.3390/diagnostics15182309

**Published:** 2025-09-11

**Authors:** Dragana Jovanovic, Rada Miskovic, Aleksandra Plavsic, Sara Radovic, Ljudmila Nagorni-Obradovic, Dragan Popovic, Milos M. Nikolic, Branka Bonaci-Nikolic

**Affiliations:** 1 Clinic of Allergy and Immunology, University Clinical Center of Serbia, 11000 Belgrade, Serbia; rada_delic@hotmail.com (R.M.); sandrony@yahoo.com (A.P.); sara.radovic21@gmail.com (S.R.); branka_bonaci@yahoo.com (B.B.-N.); 2 Faculty of Medicine, University of Belgrade, 11000 Belgrade, Serbia; ljudmila.nagorni.kcs@gmail.com (L.N.-O.); dragan.drendo23@gmail.com (D.P.); milos.nikolic@med.bg.ac.rs (M.M.N.); 3 Clinic of Pulmonology, University Clinical Center of Serbia, 11000 Belgrade, Serbia; 4 Clinic of Gastroenterohepatology, University Clinical Center of Serbia, 11000 Belgrade, Serbia; 5 Clinic of Dermatology and Venereology, University Clinical Center of Serbia, 11000 Belgrade, Serbia

**Keywords:** bactericidal/permeability-increasing (BPI) protein, BPI-ANCA, gram-negative bacteria, persistent infection, *Pseudomonas aeruginosa*, ulcerative colitis with primary sclerosing cholangitis (UC/PSC)

## Abstract

**Background/Objectives**: Binding of bactericidal/permeability-increasing (BPI) protein to Gram-negative (GN) bacteria plays a major role in bacterial elimination. The relationship between BPI-antineutrophil cytoplasmic autoantibodies (ANCA), persistent infections and immunoinflammatory diseases has not been elucidated. **Methods**: In total, 193 ANCA-positive patients detected by IIF with ANCA-associated vasculitides (AAV, n-40), connective tissue diseases (CTD, n-28), drug-induced vasculitides (DIV, n-17), ulcerative colitis (UC, n-24), UC with primary sclerosing cholangitis (UC/PSC, n-14), Crohn’s disease (CD, n-10), autoimmune hepatitis (AIH, n-19) and chronic infections (n-41) were tested using the BPI-ANCA quantitative and semiquantitative ELISA (ANCA-profile: BPI, proteinase 3, myeloperoxidase, elastase, cathepsin G, lactoferrin). BPI-ANCA were analyzed in 52 healthy persons. **Results**: A total of 46/193 (23.8%) patients had BPI-ANCA positivity. BPI-ANCA were more frequently present in patients with prolonged GN bacterial infections and inflammatory bowel diseases than in AAV, DIV, AIH, CTD and healthy controls (*p* < 0.001). UC/PSC patients more frequently had BPI-ANCA than UC and CD patients (*p* < 0.001). GN bacterial infections more frequently had BPI-ANCA than Gram-positive bacterial infections (*p* < 0.001). Infections caused by *Pseudomonas aeruginosa* and *Mycobacterium tuberculosis* had monospecific BPI-ANCA (sensitivity 79% and 71%, respectively). UC/PSC and chronic GN bacterial infections caused by *Klebsiella pneumoniae*, *Proteus mirabilis*, or *Escherichia coli* had multispecific BPI-ANCA (sensitivity 64% and 100%, respectively). Odds ratio analysis showed that patients with IBD who were positive for multispecific BPI-ANCA had a 13.5-fold increased risk of UC/PSC (95% CI 2.98–61.18). **Conclusions**: Monospecific BPI-ANCA may be a valuable biomarker for persistent *Pseudomonas aeruginosa* and *Mycobacterium tuberculosis* infections. In contrast, multispecific BPI-ANCA are associated with UC/PSC and persistent infections caused by intestinal Gram-negative bacteria. Suppression of antimicrobial function by multispecific BPI-ANCA could impair the elimination of Gram-negative bacteria, sustaining the immunoinflammation. Dysregulated antimicrobial response might be the target of immunomodulatory therapy in the initial phase of BPI-ANCA-positive UC/PSC.

## 1. Introduction

Endogenous bactericidal permeability-increasing protein (BPI) is a multifunctional cationic protein produced by neutrophils and macrophages that has high antibacterial and anti-inflammatory properties. BPI protein, with two functionally important domains, is a host protein that binds to endotoxins and plays an important role in defense against Gram-negative (GN) bacteria. Endotoxins are unique glycolipids, mainly the lipopolysaccharide (LPS) found in the outer membrane of GN bacteria. The cytotoxic function of the BPI protein is to neutralize endotoxins via its N-terminal domain, while the C-terminal domain of BPI disables LPS delivery to CD14 and thus inhibits CD14/myeloid differentiation factor (MD)-2/toll-like receptor (TLR) 4-dependent endotoxin-mediated cell activation [1]. BPI protein competes with lipoprotein binding protein (LBP) for LPS. LBP-LPS interaction is required for LPS recognition by TLR4 and MD-2 receptors to induce an inflammatory response [1,2]. BPI protein has an opsonizing effect through the N- and C-terminal parts, which facilitates phagocytosis and contributes to the presentation of antigens to immune cells. The BPI protein, as a central component of the innate immune system, modulates adaptive immune responses, so deregulation of the antimicrobial response can potentially contribute to immunoinflammatory diseases [2].

According to the literature, BPI protein has been repeatedly confirmed to be a target for antineutrophil cytoplasmic autoantibodies (ANCA). These ANCA directed against BPI proteins (BPI-ANCA) have been identified in several inflammatory disorders and infectious diseases, such as inflammatory bowel diseases (IBD), chronic obstructive pulmonary disease (COPD), non-cystic fibrosis (CF) bronchiectasis, tuberculosis, and cystic fibrosis associated with *Pseudomonas aeruginosa* (*P. aeruginosa*) infection. BPI-ANCA prevents the BPI-induced killing of *P. aeruginosa*. However, little is known about the role of BPI protein in Gram-positive (GP) bacterial infections and tuberculosis [2,3]. In human pathology, the interaction between microbial infection and host immunity can contribute to a variety of diseases. It has been demonstrated that a dysfunctional, excessive inflammatory immune response combined with chronic pulmonary GN infections destroys the airways, leading to morbidity and even mortality [4]. There are several hypotheses explaining the pathophysiological events that lead to tissue damage. Numerous studies attempted to elucidate the specific host–pathogen interactions during chronic infections caused by opportunistic GN pathogens [4]. However, the relationship between BPI-ANCA in persistent infections and immunoinflammatory diseases has not yet been elucidated. Given the strong association between persistent GN infections and BPI-ANCA, it is possible that these antibodies allow the bacteria to evade the immune response, contributing to the worsening of infection and induction of chronic immunoinflammatory diseases.

In order to examine the potential diagnostic significance of BPI-ANCA in different chronic infections and immunoinflammatory diseases, we analyzed the prevalence and associations of BPI-ANCA and other ANCA auto-antigens in persistent bacterial and viral infections, ulcerative colitis (UC), UC with primary sclerosing cholangitis (PSC), Crohn’s disease (CD), autoimmune hepatitis (AIH), drug-induced vasculitides (DIV), ANCA-associated vasculitides (AAV), and connective tissue diseases (CTD). We also investigated the association of BPI-ANCA with other ANCA autoantigens: myeloperoxidase (MPO), proteinase 3 (PR3), lactoferrin (Lf), cathepsin G (Cat-G), and leukocyte elastase (LE). According to these results, we studied whether monospecific BPI-ANCA and multispecific BPI-ANCA could be a useful serological marker for persistent infections and immunoinflammatory diseases.

## 2. Materials and Methods

### 2.1. Patient Selection

We evaluated the presence of ANCA by indirect immunofluorescence (IIF) in 15,112 sera sent to the Laboratory for Allergy and Clinical Immunology at the University Clinical Center of Serbia in Belgrade during the period of five years.

Their immunological profile was studied because of various clinical and laboratory disorders, such as elevated acute phase reactants, hypochromic anemia, altered hepatogram, prolonged fever, weight loss, myalgia, arthralgia/arthritis, rash, persistent diarrhea, jaundice, and multi-organ involvement. Inclusion criteria for the study were as follows: patients with confirmed persistent infections treated only with antimicrobial therapy according to the antibiogram and newly diagnosed patients with confirmed UC, UC/PSC, CD, AIH, DIV, AAV, and CTD. Patients were not previously treated with immunosuppressive therapy (serum samples were analyzed at the time of diagnosis). The exclusion criterion was the presence of associated malignant disease.

According to the criteria [5,6,7,8,9], 41 patients were diagnosed with persistent infections caused by *P. aeruginosa, Klebsiella pneumoniae* (*Klebsiella*), *Proteus mirabilis* (*P.mirabilis*), *Echerichia coli* (*E. coli*), *Streptococcus pyogenes*, *Staphylococcus aureus*, *Streptococcus pneumoniae*, *Mycobacterium tuberculosis*, and hepatitis C virus (HCV). According to the criteria [10,11,12,13,14,15], 152 patients had chronic immunoinflammatory diseases; 48 patients had IBD, 19 AIH, 40 AAV, 17 DIV (13 propylthiouracil and 4 methimazole-induced AAV), and 28 CTD [13,14,15]. The disease diagnoses and main demographic characteristics of the 193 ANCA-positive patients detected by IIF in our study group are shown in Table 1. The control group consisted of 52 blood donors who were matched by gender and age with the study group.

In total, 193/15,112 (1.2%) consecutive ANCA-positive patients detected by IIF were studied for the presence, concentration, and association of BPI-ANCA with other ANCA autoantigens using quantitative and semiquantitative (ANCA profile) enzyme-linked immunosorbent assay (ELISA).

A total of 46/193 (23.8%) BPI-ANCA-positive patients were divided into two groups. The first group consisted of 24/46 (52.2%) patients with ANCA positivity exclusively against the BPI antigen (monospecific BPI-ANCA). The second group comprised 22/46 (47.8%) patients with ANCA positivity against multiple ANCA antigens, including the mandatory BPI antigen (multispecific BPI-ANCA).

### 2.2. IIF for Detection of ANCA

The presence, titers, and type of IgG-ANCA immunofluorescence were determined by IIF assay at the first visit with ethanol-fixed human neutrophils, according to the manufacturer’s instructions (EUROIMMUN, Lübeck, Germany). For all patients, sera were diluted to an initial titer of 1:80 in phosphate-buffered saline containing 0.2% bovine serum albumin. Samples were incubated for 60 min at room temperature and then washed three times with wash buffer. Anti-human IgG antibodies labeled with FITC were added and incubated for 30 min at room temperature. After washing, samples were analyzed using a fluorescence microscope with incident light illumination (EUROStar III Plus, IFA diagnostics, Euroimmun) at 400× and 1000× magnifications. ANCA were classified as follows: atypical xANCA (inhomogeneous, mild, or strong rim staining of the nuclear periphery), cytoplasmic cANCA (diffuse cytoplasmic granular staining with lobular accentuation), and perinuclear pANCA (perinuclear homogeneous staining). Positive samples were double diluted to determine the ANCA titer.

### 2.3. ANCA-Profile

Serum samples were analyzed for ANCA using the ELISA test method for six native neutrophil antigens: BPI, PR3, MPO, EL, Cat-G, and Lf (ANCA Profile ELISA IgG, EUROIMMUN, Lübeck, Germany). The assays were performed according to the manufacturer’s instructions. Samples were diluted 1:101 and added to the wells of the plate. These samples were incubated at room temperature for 30 min and then washed three times with the wash buffer. Then, an enzyme conjugate containing horseradish peroxidase (HRP)-labeled anti-human IgG antibodies was added to the wells, and the wells were incubated for 30 min at room temperature. After washing with the same wash buffer, the substrate TMB (3.3′, 5.5′-tetramethylbenzidine) was added to the wells for 15 min to react with the HRP and allow for a measurable color change. After washing, the reaction was stopped using a stop solution that contained acid. The optical density was measured at 450 nm (reference 650 nm) using a microplate reader (Rayto RT-6100, Shenzhen, China). According to the manufacturer’s recommendations, samples with optical densities (OD) greater than the OD of the cut-off control serum were considered positive. For PR3, the calculated ratio was multiplied by the factor 1.4. The ratio values (sample/cut-off) for each antigen were in the following range: 1–2 weakly positive (+); 2–5 positive (++); >5 highly positive (+++).

### 2.4. Concentration of BPI-ANCA

The specificity of BPI-ANCA (cut-off value 10 U/mL) was also confirmed by a direct ELISA for quantitative determination of IgG-BPI-ANCA according to the manufacturer’s instructions. (Organtec Diagnostic, GmbH, Mainz, Germany). A total of 10 μL of undiluted sample was added to the bottom of the well. The method was performed in the same way as the determination of ANCA-antigen specificity (ANCA-profile) by ELISA, with the difference that this test included a standard curve and the second incubation lasted 15 min. Also, the optical density was measured at 450 nm within 30 min after the addition of the stop solution.

### 2.5. Statistics

The obtained data were analyzed using IBM SPSS Statistics software for Windows (version 26; IMB, Armonk, NY, USA). Mean values of quantitative variables were used, and frequencies of qualitative variables were calculated. The nonparametric Chi-squared and Fisher’s exact tests were used to assess relationships between the qualitative variables. The Wilcoxon signed-rank test and the *t*-test were used to compare two paired samples. A *p* value ≤ 0.05 was considered significant. According to the confirmed diagnosis of BPI-ANCA-positive patients, the sensitivity, specificity, and positive predictive value were calculated using the following formulas: sensitivity = true positive/true positive + false negative × 100%, specificity = true negative/true negative + false positive × 100%, and positive predictive value = true positive/true positive + false positive × 100%. To estimate the probability of UC/PSC in patients with IBD, the odds ratio (OR) of multispecific BPI-ANCA was calculated. Receiver operating characteristic (ROC) curve analysis was performed to identify the optimal concentration threshold of multispecific BPI-ANCA to distinguish UC/PSC from UC.

## 3. Results

### 3.1. General Characteristics of Patients

Our study group included 121 female and 72 male patients with a mean age of 45.76 ± 15.97 (Table 1). A total of 46/193 (23.8%) patients were found to be BPI-ANCA-positive using ANCA-profile (ELISA) and quantitative BPI-ANCA ELISA. Monospecific BPI-ANCA was present in 24/46 (52.2%) BPI-ANCA-positive patients, while multispecific BPI-ANCA was present in 22/46 (47.8%) BPI-ANCA-positive patients. There is no difference in gender and age between monospecific and multispecific BPI-ANCA-positive patients (Table 2). All 52 healthy controls were BPI-ANCA negative.

We found that 11/46 (24%) BPI-ANCA-positive patients had *P. aeruginosa* infection, 6/46 (13%) patients had other GN bacterial infections (*Klebsiella*, *P. mirabilis,* or *E. coli*), 5/46 (11%) had *Mycobacterium tuberculosis* infections, 8/46 (19.6%) had UC, and 10/46 (21.7%) had UC/PSC. Only 3/46 BPI–ANCA-positive patients had AIH, 2/46 had DIV, and 1/46 had AAV. BPI-ANCA positivity was more frequently observed in patients with prolonged GN bacterial or *Mycobacterium tuberculosis* infections (22/46, 48%) and in those with UC or UC/PSC (18/46, 39.1%) compared with the remaining BPI-ANCA-positive patients (*p* < 0.001).

All BPI-ANCA-positive patients in the ANCA profile were positive in quantitative BPI-ANCA ELISA. The mean BPI-ANCA concentration was significantly higher in patients with monospecific BPI-ANCA (64.3 U/mL) compared with those with multispecific BPI-ANCA (32.4 U/mL) (*p* = 0.036) (Table 2).

#### 3.1.1. Type of IIF and Titer of ANCA in BPI-ANCA-Positive Patients

**Figure 1:**
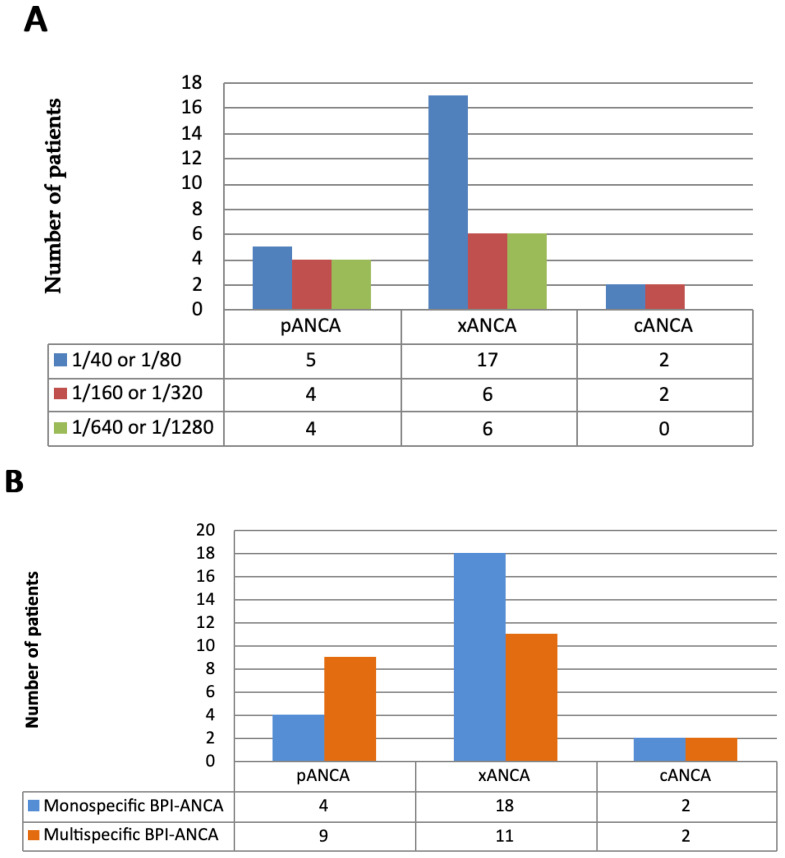
(**A**) ANCA type and ANCA titer detected by IIF in 46 BPI-ANCA-positive patients; (**B**) ANCA type in monospecific BPI-ANCA-positive and multispecific BPI-ANCA-positive patients.

Among patients with BPI-ANCA-positivity, presence of xANCA was more frequently detected (29/46, 63%) than pANCA (13/46, 28.2%) and cANCA (4/46, 8.7%) (*p* < 0.01). Titers of xANCA in BPI-ANCA-positive patients ranged from 1:40 to 1:640, with a median titer of 1/160 (Figure 1A). Monospecific BPI-ANCA was most often associated with xANCA, whereas multispecific BPI-ANCA showed similar frequencies of pANCA and xANCA (Figure 1B).

#### 3.1.2. BPI-ANCA and ANCA Profile in Patients with Prolonged Infections

Patients with persistent infections had significantly higher incidence and level of monospecific BPI-ANCA than multispecific BPI-ANCA (*p* < 0.05). Among patients with persistent infections, BPI-ANCA positivity was found only in patients with GN bacterial infections (17/20, 85%) and tuberculosis (5/7, 71.4%), while patients with GP bacterial infections (0/9, (*p* < 0.001)) and HCV infections (0/5, (*p* < 0.001)) were BPI-ANCA negative (Table 2).

Patients with bacterial infections caused by *P. aeruginosa* and *Mycobacterium tuberculosis* had only monospecific BPI-ANCA, with a high concentration of BPI-ANCA (68.6 U/mL and 70.2 U/mL, respectively).

Patients with GN bacterial infections caused by *Klebsiella*, *P. mirabilis,* or *E coli* had only multispecific BPI-ANCA (100%) with a high concentration of BPI-ANCA (78.0 U/mL) (Table 2 and Table 3).

The specificities and level of positivity for various ANCA antigens (Table 3) were tested for 46 BPI-ANCA-positive samples. The most frequently observed specificities in patients with GN bacterial infections caused by *Klebsiella*, *P. mirabilis,* or *E. coli* were PR3-ANCA and MPO-ANCA, with predominantly medium and low concentrations (Table 3).

#### 3.1.3. BPI-ANCA and ANCA Profile in IBD Patients

In the IBD group of patients, positive BPI-ANCA were more frequently detected in UC and UC/PSC patients (18/38, 47.4%) than in CD patients (0/10) (*p* < 0.001).

UC/PSC patients more frequently had positive BPI-ANCA 10/14 (71.4%) compared with UC patients (8/24, 33.3%) (*p* = 0.042) (Table 2).

Patients with UC/PSC had significantly higher incidence of multispecific BPI-ANCA than monospecific BPI-ANCA (*p* < 0.01). In the group of patients with multispecific BPI-ANCA, those with UC/PSC (9/14, 64.2%) more frequently had multispecific BPI-ANCA compared with UC patients (4/24, 16.7%) (*p* < 0.01). Moreover, UC/PSC patients had a higher mean concentration of BPI-ANCA (35.6 U/mL) compared with UC patients (19.5 U/mL) (*p* = 0.017) (Table 2).

The most frequently associated ANCA specificity in UC/PSC patients was PR3 (Table 3). Patients with UC/PSC more frequently had ANCA specific to BPI and PR3 (*p* < 0.01) compared with UC patients.

#### 3.1.4. BPI-ANCA and ANCA Profile in Patients with AIH, DIV, AAV, and CTD

The minority of patients with AIH 3/19 (15.8%), DIV 2/17 (11.7), and AAV 1/40 (2.5%) were BPI-ANCA positive. CTD patients were BPI negative. Two patients with DIV had multispecific BPI-ANCA. The most frequently observed associated specificities in these patients were PR3 and MPO (Table 3).

#### 3.1.5. Sensitivity, Specificity, and Positive Predictive Value of Monospecific and Multispecific BPI-ANCA in Patients with Persistent Infections and UC/PSC

**Figure 2:**
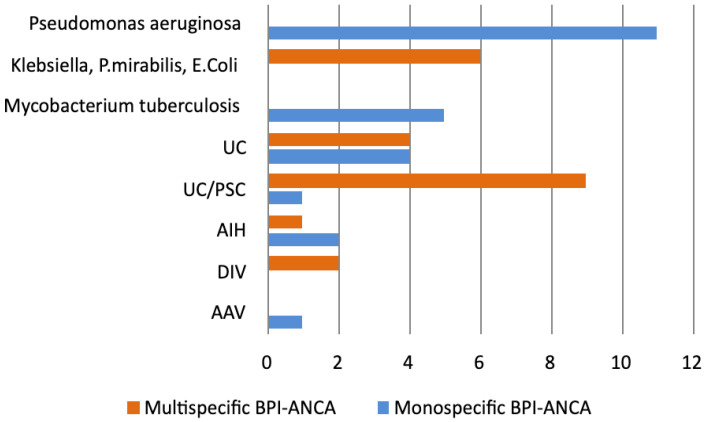
The frequency of monospecific BPI-ANCA and multispecific BPI-ANCA in 46 BPI-ANCA-positive patients. *Klebsiella pneumoniae*: *Klebsiella*, *Proteus mirbilis*: *P*. *mirabilis*, *Echerichia coli*: *E.coli*. UC: Ulcerative colitis; UC/PSC: Ulcerative colitis with primary sclerosing cholangitis; AIH: Autoimmune hepatitis; DIV: Drug-induced vasculitides; AAV: ANCA-associated vasculitides.

Patients with chronic *P. aeruginosa* and *Mycobacterium tuberculosis* infections had exclusively monospecific BPI-ANCA (Figure 2). Monospecific BPI-ANCA had a high sensitivity and specificity for *P. aeruginosa* infection (79% and 80%, respectively) and tuberculosis (71% and 77. 9%, respectively) (Table 4). In patients with chronic infections, monospecific BPI-ANCA had a high sensitivity, specificity, and positive predictive value for *P. aeruginosa* or *Mycobacterium tuberculosis* infections (76.2%, 100%, and 100%, respectively).

On the contrary, multispecific BPI-ANCA was more frequently observed in GN bacterial infection (*Klebsiella*, *P. mirabilis,* or *E. coli*) and UC/PSC (Figure 2). In ANCA-positive patients, the sensitivity and specificity of multispecific BPI-ANCA for GN bacterial infection (*Klebsiella*, *P. mirabilis*, or *E. coli*) were 100% and 78.6%, respectively, and for UC/PSC, they were 64.3% and 79.9%, respectively (Table 4). In a group of IBD patients, multispecific BPI-ANCA had sensitivity, specificity, and positive predictive value for UC/PSC (64.3%, 88%, and 69.2%, respectively). Odds ratio analysis showed that patients with IBD who were positive for multispecific BPI-ANCA had a 13.5-fold increased risk of UC/PSC (95% CI 2.98–61.18).

#### 3.1.6. ROC Curve Analysis of Multispecific BPI-ANCA for Distinguishing UC/PSC from UC

**Figure 3:**
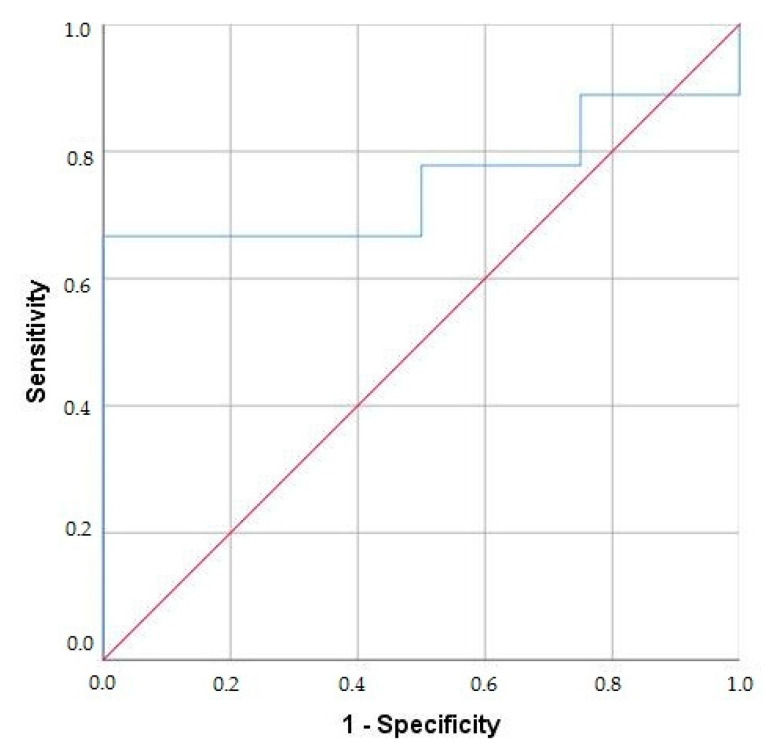
ROC curve (the blue line) for cut-off concentration of the multispecific BPI-ANCA to distinguish UC/PSC from UC (AUC 0.75; CI 0.482–1.0). The diagonal of chance (the red line) distinguishes patients with and without disease..

ROC curve analysis was performed to identify the optimal threshold of multispecific BPI-ANCA concentration to distinguish UC/PSC from UC. A BPI-ANCA concentration of 18.9 U/mL differentiated UC/PSC from UC patients with an AUC of 0.75, a sensitivity of 77.8%, and a specificity of 50% (95% CI 0.482–1.0) (Figure 3).

## 4. Discussion

This study investigated the presence of monospecific and multispecific BPI-ANCA in a large number of ANCA-positive patients with various persistent infections and immunoinflammatory diseases.

Although IIF represents the first level for ANCA detection, ANCA profile (ELISA) is the most important diagnostic step for detection of ANCA specific to several neutrophil antigens (BPI, PR3, MPO, EL, Cat-G, and Lf). In our study, monospecific or multispecific BPI-ANCA were associated with prolonged GN bacterial infections, tuberculosis, and UC/PSC. Suppression of antimicrobial function by BPI-ANCA could impair the local elimination of GN bacteria, thereby sustaining chronic immunoinflammation.

Our study demonstrates the potential diagnostic significance of BPI-ANCA positivity in patients with prolonged bacterial infections and UC/PSC in clinical practice. We have found that monospecific BPI-ANCA may be a useful marker for persistent *Pseudomonas aeruginosa* and *Mycobacterium tuberculosis* infections. On the contrary, multispecific BPI-ANCA were associated with UC/PSC and persistent infections with GN bacteria, which colonize the alimentary tract. Furthermore, BPI-ANCA levels were significantly higher in the group of patients with monospecific BPI-ANCA than in the group with multispecific BPI-ANCA.

According to the findings of our study, 46 out of 193 patients were BPI-ANCA positive, whereas none of the healthy controls and CTD patients tested positive. The majority of BPI-ANCA-positive patients had prolonged bacterial infections (22/46) or UC and UC/PSH (18/46). Other patient groups showed a significantly lower percentage of BPI-ANCA positivity, including AIH (3/46), DIV (2/46), and AAV (1/46).

The majority of patients with BPI-ANCA positivity had atypical perinuclear ANCA (xANCA) 29/46 (63%). ANCA are a group of autoantibodies associated with various immunoinflammatory diseases and induce distinct fluorescence patterns. Primary vasculitides are associated with cytoplasmic staining targeting PR3 (cANCA), typical for granulomatosis with polyangiitis (GPA), and perinuclear staining targeting MPO (pANCA), typical for microscopic polyangiitis (MPA) [16,17]. xANCA that we found in the majority of our BPI-ANCA-positive patients is often seen in IBD patients [18,19,20].

The pathogenesis of IBD is not yet fully understood, but an abnormal immune response to dysbiosis of the gastrointestinal microbiome in genetically susceptible individuals plays an important role. Biomarkers associated with dysbiosis and mucosal healing are the subject of numerous investigations in IBD [21]. ANCA and anti-Saccharomyces cerevisiae antibodies (ASCA), directed against the gastrointestinal microbiome, are believed to contribute to inflammation in IBD patients [22]. The predictive value of a xANCA-negative/ASCA-positive result is 95% for CD, while a xANCA-positive/ASCA-negative result is 90% predictive for UC [23]. The presence of various antibodies in IBD patients implies dysregulated immune response [21,23].

It is well established that the gastrointestinal microbiome plays an important role in the development of the immune system. The composition of the gastrointestinal microbiome is diverse and includes a variety of microbes, with *Firmicutes*, *Bacteroidetes*, and *Actinobacteria* being among the most dominant, while *E. coli* and *Lactobacillus* are present to a lesser extent. Intestinal dysbiosis can lead not only to gastrointestinal disorders, but also to dysfunctions in other distal organs and systems [22,24]. Dysbiosis of the gastrointestinal microbiome, together with dysregulated innate and adaptive immune responses influenced by genetic susceptibility, contributes to persistent intestinal inflammation and tissue damage in IBD. A reduced response to LPS enhances survival of certain GN bacteria, promoting their translocation into the intestinal epithelium and triggering inflammation. Dysregulated cytokine production recruits additional inflammatory cells, thereby perpetuating the immune-mediated inflammatory response. [24].

ANCA specific for BPI proteins and for other various antigens, predominantly PR3 and MPO, may indicate ongoing inflammation in patients with IBD and AIH [16,25,26,27]. Our previously published study demonstrated that the BPI protein is the most important target antigen in ANCA-positive UC and UC/PSC patients [20]. BPI proteins are present in significant concentrations in the intestinal mucosa of patients with IBD [28]. Interestingly, we also did not find BPI-ANCA positivity in CD patients. Our current results confirm that more than 70% UC and UC/PSC patients had multispecific BPI-ANCA. Furthermore, we confirmed previously published data showing that BPI-ANCA are most commonly associated with PR3-ANCA in UC/PSC patients [26]. Patients with UC/PSC also exhibited higher BPI-ANCA levels compared with UC patients [26]. We have also found higher concentrations of BPI-ANCA in UC/PSC in comparison with UC patients. In our study, patients with UC/PSC had a significantly higher incidence of multispecific BPI-ANCA than monospecific BPI-ANCA. Also, the finding of multispecific BPI-ANCA was significantly higher in the group of patients with UC/PSC than in the group of UC patients (64.3% and 16.7%, respectively). We showed that multispecific BPI-ANCA had a positive predictive value of 69.2% and a high OR (13.5) for UC/PSC in patients with IBD. A significantly higher level of multispecific BPI-ANCA was found in patients with UC/PSC (35.6 U/mL) than in patients with UC (19.5 U/mL). We also demonstrated that a BPI-ANCA concentration of 18.9 U/mL distinguishes UC/PSC from UC (AUC 0.75). The most commonly observed associated specificity in these patients was PR3, which occurred at a significantly higher frequency in UC/PSC patients compared with UC patients (57% and 13%, respectively).

It is widely recognized that PR3-ANCA, which is an established marker for GPA [16], is found in certain groups of patients with more severe forms of UC and UC/PSH [20,29,30]. The possibility of a pathogenic association between AAV and IBD should not be excluded [29]. It has been confirmed that GPA and CD are characterized by granulomatous inflammation mediated by Th1 and Th17 cells, suggesting a similar role for lymphocytes in granulomatous inflammation [31]. On the other hand, eosinophilic granulomatosis with polyangiitis and UC are Th2-mediated diseases, suggesting a role for eosinophils in inflammation [29]. Likewise, patients with high PR3-ANCA titers and overlapping UC or CD and GPA have been reported [31]. However, the cause-and-effect relationship between these two diseases and their mutual influence remains unclear.

We already know that intestinal dysbiosis may lead to disruption of the natural intestinal barrier, damage to the intestine, and a consequent increase in intestinal permeability to various bacterial endotoxins [32]. BPI-ANCA contributes to inflammation caused mainly by GN bacteria by reducing the natural antibacterial ability of BPI [16,20,28]. A recent study showed that positive BPI-ANCA had a high positive predictive value for bacterial infection in AAV [33]. A link between infection and vasculitis has long been recognized. For example, associations between hepatitis B virus infection (HBV) and polyarteritis nodosa or HCV infection and cryoglobulinemic vasculitis are now well established [34]. Increasing evidence indicates that the gastrointestinal microbiome and neutrophil antigens, especially BPI, play a significant role in the development of various immunoinflammatory diseases [35,36].

We also demonstrated BPI-ANCA positivity in a significant proportion of patients with prolonged GN bacterial infections, with 17 out of 20 patients (85%) testing positive. Our data showed that patients with prolonged *P. aeruginosa* infection had only monospecific BPI-ANCA. Among them, three patients had CF, five patients had bronchiectasis, and three had COPD. According to our results, the identification of monospecific BPI-ANCA in patients with undiagnosed lung symptomatology may help in the diagnosis of CF, bronchiectasis, or COPD associated with prolonged *P. aeruginosa* infection. According to the literature, BPI-ANCA positivity is often present in the serum of patients with CF and COPD, and the level of BPI-ANCA correlates to airway colonization with *P. aeruginosa* and pulmonary exacerbations [3,4].

Previous studies have shown that BPI protein exhibits a strong antimicrobial effect against GN bacteria, including *P. aeruginosa*, contributing to the opsonization and neutralization of GN bacteria endotoxin [37]. BPI protein is one of the most powerful natural antibiotics produced by neutrophils and exhibits a high affinity for the LPS of GN bacterial cell membranes. Binding of BPI to the lipid A portion of LPS of GN bacteria leads to their elimination, inhibition of the pro-inflammatory cytokine secretion, including tumor necrosis factor α, interleukin 6, and interferon γ, as well as suppression of complement activation and the fibrinolytic pathway, collectively preventing further inflammation [15]. Commensal and symbiotic interactions of many GN bacteria, which are present along the different mucosal surfaces, are important for normal development of the immune system and maintenance of homeostasis [32,38]. The host’s response to the endotoxin in the tissue can result in both protective inflammatory responses and systemic pathological reactions [39]. Epitope mapping of BPI-ANCA-positive patients revealed a high similarity of the BPI protein epitopes and the outer membrane proteins of *E. coli* and *P. aeruginosa* [40]. Therefore, molecular mimicry may be one of the mechanisms driving BPI-ANCA development. The process by which infection induces BPI-ANCA remains unclear; however, one hypothesis is that persistent GN bacterial infection and endotoxin exposure, along with local accumulation of neutrophils in neutrophil extracellular traps and prolonged delivery of opsonized GN bacteria (coated with BPI) to dendritic cells, may lead to BPI-ANCA induction. Furthermore, abundant and/or persistent accumulation of endotoxin may overwhelm the capacity of his neutralization, leading to a breakdown of tolerance and consequent BPI-ANCA induction [2].

In accordance with this, the highest sensitivity of up to 100% was observed for heterospecific BPI-ANCA in our patients with prolonged GN bacterial infections caused by *Klebsiella*, *P. mirabilis,* or *E. coli*. We also noticed that the most commonly observed associated specificities in these patients were PR3 and MPO, with a significant frequency of 100% and 50%, respectively.

Interestingly, among our patients with prolonged infections caused by *Klebsiella*, *P. mirabilis,* or *E. coli*, there were three patients with retroperitoneal fibrosis (RPF). These three patients experienced prolonged urinary tract infection caused by *Klebsiella* or *E coli*. RPF can be classified as an idiopathic fibroinflammatory disease involving the abdominal aorta, iliac arteries, and retroperitoneum or as secondary RPF, most commonly resulting from persistent infection [41]. It has been shown that one-third of patients with RPF are ANCA-positive, the most frequent being PR3-ANCA [42]. However, we are the first to demonstrate the presence of a multispecific BPI-ANCA targeting both BPI and PR3 simultaneously in our three patients.

We have demonstrated monospecific BPI-ANCA in five of seven patients with pulmonary tuberculosis (71.4%). *Mycobacterium tuberculosis* is an intracellular pathogenic microorganism that primarily causes pulmonary infection in individuals with weakened immune systems. Previous studies have shown that patients with tuberculosis infection have significantly increased concentrations of BPI-ANCA, which may contribute to organ damage [2,43,44]. This finding can be explained by the fact that the BPI protein has direct bactericidal effects on mycobacteria and indirectly by modulating macrophage responses. In both mechanisms, inhibition of *Mycobacterium tuberculosis* growth and stimulation of the immune response in human macrophages contribute to a strong potential for combating tuberculosis [44]. Human BPI protein is mainly produced by neutrophils for bactericidal function, but it is also possible that BPI protein can be internalized by macrophages for the control of intracellular bacteria [45]. Macrophages can activate different mechanisms for the internalization of BPI that are involved in protein transport into human cells via endocytosis, pinocytosis, or direct membrane penetration through pore or micelle formation. Likewise, recombinant BPI protein was found to significantly reduce intracellular growth of avirulent and virulent strains of *Mycobacterium tuberculosis* without affecting macrophage viability [44]. BPI protein can also damage the surface of mycobacteria and partially increase membrane permeability. Lipoarabinomannan (LAM), a glycolipid from the cell wall of *Mycobacterium*, has a similar chemical structure to LPS and can bind to the CD14 receptor in the same way as LPS [46]. In this context, BPI protein may interact with mycobacterial LAM, promoting phagocytosis, inhibiting the production of TNF-α, and causing an anti-inflammatory effect, similar to that observed in GN bacterial infections. The presence of multispecific BPI-ANCA in patients with primary or secondary ANCA vasculitides, especially following initiation of immunosuppressive therapy, should raise the suspicion of concomitant tuberculosis [25,47,48].

While our study combined IIF, ELISA (ANCA-profile), and quantitative BPI-ANCA, one limitation was the lack of ELISA assessment of the other neutrophil antigens, including proteinase 3, myeloperoxidase, cathepsin G, lactoferrin, and esterase. Although the initial number of tested patients was large (N 15,112), the number of ANCA-positive patients with certain infections, such as *Mycobacterium tuberculosis*, was relatively small. Additionally, the proposed link between intestinal dysbiosis, genetic susceptibility, and BPI-ANCA formation is plausible but remains uninvestigated. Despite these limitations, our results highlight the clinical significance of monospecific and multispecific BPI-ANCA findings in persistent infections and various immunoinflammatory diseases. Clinical validation of our results will require further multicenter studies. Further research, including microbiome sequencing, would yield important information and could provide directions for further research.

## 5. Conclusions

Our results demonstrate that, in ANCA-positive patients diagnosed by IIF, monospecific BPI-ANCA may serve as a useful biomarker for chronic *Pseudomonas aeruginosa* and *Mycobacterium tuberculosis* infections. In contrast, multispecific BPI-ANCA may be a valuable biomarker for UC/PSC and persistent infections caused by GN bacteria, such as *Klebsiella pneumoniae*, *Proteus mirabilis*, or *Escherichia coli*, that colonize the gastrointestinal tract. Suppression of antimicrobial function by multispecific BPI-ANCA could impair the local elimination of GN bacteria, sustaining the chronic immunoinflammation. BPI-ANCA decreases innate antimicrobial response and contributes to prolonged GN bacterial infections and the development of chronic immunoinflammatory response typical for UC/PSC. Dysregulation of BPI protein function and high concentration of ANCA against BPI, which we found in UC/PSH, suggest an important influence of prolonged GN bacterial stimulation on the break of tolerance and might be targets for future, early immunomodulatory therapy in the initial phase of UC/PSC.

## Figures and Tables

**Table 1:** Diagnoses and main demographic characteristics of 193 ANCA-positive (IIF) patients.

	ANCA-Positive (IIF) Patients *n* = 193
Age, years (MV ± SD) Years range	45.76 ± 15.97 18–80
Male sex, *n* (%)	121 (62.7)
Female sex, *n* (%)	72 (37.3)
Persistent bacterial and viral infections, *n* (%)	41 (21.2)
*Pseudomonas aeruginosa*, *n* (%)	14 (7.3)
- Cystic fibrosis	3
- Bronchiectasis	5
- Chronic obstructive pulmonary disease	6
*Klebsiella pneumoniae*, *Proteus mirabilis* or *Echerichia coli*, *n* (%)	6 (3.1)
- Urinary tract infection (UTI)	3
- UTI complicated by retroperitoneal fibrosis	3
*Mycobacterium tuberculosis*, *n* (%)	7 (3.6)
*Streptococcus pyogenes*, *Staphylococcus aureus* or *Streptococcus pneumoniae*, *n* (%)	9 (4.7)
- Empyema	2
- Chronic obstructive pulmonary disease	5
- Chronic sinusitis	2
*Hepatitis C Virus*, *n* (%)	5 (2.6)
Inflammatory bowel diseases, *n* (%)	48 (24.9)
Ulcerative colitis, *n* (%)	24 (12.4)
Ulcerative colitis with primary sclerosing cholangitis, *n* (%)	14 (7.3)
Crohn’s disease, *n* (%)	10 (5.2)
Autoimmune hepatitis, *n* (%)	19 (9.8)
Vasculitides, *n* (%)	57 (29.5)
Drug-induced vasculitides, *n* (%)	17 (8.8)
ANCA-associated vasculitides, *n* (%)	40 (20.7)
- Granulomatosis with polyangiitis	16 (8.3)
- Microscopic polyangiitis	20 (10.4)
- Eosinophilic granulomatosis with polyangiitis	4 (2.1)
Connective tissue diseases, *n* (%)	28 (14.5)
- Systemic lupus erythematosus, *n* (%)	14 (7.3)
- Sjögren syndrome, *n* (%)	10 (5.2)
- Systemic sclerosis, *n* (%)	4 (2.1)

**Table 2:** Prevalence and levels of monospecific, multispecific, and negative BPI-ANCA in 193 ANCA-positive patients with persistent infections and immunoinflammatory diseases.

	Monospecific BPI-ANCA + *n* = 24	Multispecific BPI-ANCA + *n* = 22	Negative BPI-ANCA *n* = 147
Age, years range (MV ± SD)	49.6 ± 17.7	39.5 ± 13.9	45.5 ± 17.6
Male sex, *n* = 121	9	8	55
(%)	50.7 ± 19.0	36.5 ± 13.4	45.6 ± 18.4
Female sex, *n* = 72	15	14	92
(%)	48.7 ± 16.4	42.4 ± 14.7	45.3 ± 16.6
Persistent infections, *n* = 41	16 (66.7) *	6 (27.3)	19 (12.9)
BPI-ANCA (MV ± SD) U/mL	69.4 ± 23.4 **	28.1 ± 2.6	3.5 ± 2.9
Gram-negative bacterial infections, *n* = 20 (%)	11 (45.8)	6 (27.3)	3 (2.0)
* Pseudomonas aeruginosa*, *n* = 14 (%)	11 (45.8) **	0	3 (2.0)
BPI-ANCA (MV ± SD) U/mL	68.6 ± 28.1 **		(3.0 ± 2.8)
* Klebsiella pneumoniae*, *Proteus mirabilis*, or *Echerichia coli*, *n* = 6 (%)	0	6 (27.3) **	0
BPI-ANCA (MV ± SD)U/mL		78.0 ± 25.7 **	
*Mycobacterium tuberculosis*, *n* = 7 (%)	5 (20.8) *	0	2 (1.7)
BPI-ANCA (MV ± SD) U/mL	70.2 ± 18.8 **		(2.8 ± 3.1)
Gram-positive bacterial infections, *n* = 9 (%)	0	0	9 (6.1)
* Streptococcus pyogenes*, *Staphylococcus aureus* or			
* Streptococcus pneumoniae*, *n* = 9 (%)	0	0	9 (6.1)
BPI-ANCA (MV ± SD) U/mL			(4.3 ± 3.1)
Viral infections, *n* = 5	0	0	5 (3.4)
Hepatitis C Virus, *n* = 5 (%)	0	0	5 (3.4)
BPI-ANCA (MV ± SD) U/mL			(3.42 ± 2.9)
Inflammatory bowel diseases, *n* = 48 (%)	5 (20.1)	13 (59.1) *	30(20.4)
BPI-ANCA (MV ± SD) U/mL	47 ± 15.4	27.6 ± 15.2	(3.1 ± 4.4)
UC, *n* = 24 (%)	4 (16.7)	4 (18.2)	16 (10.9)
BPI-ANCA (MV ± SD) U/mL	44.8 ± 15.9 **	19.5 ± 17.6	(3.3 ± 3.0)
UC/PSC, *n* = 14 (%)	1 (4.2)	9 (40.9) **	4 (2.7)
BPI-ANCA (MV ± SD) U/mL	49.2 ± 14.9	35.6 ± 12.7	(3.8 ± 5.5)
CD, *n* = 10 (%)	0	0	10 (11)
BPI-ANCA (MV ± SD) U/mL			(2.1 ± 4.4)
AIH, *n* = 19 (%)	2 (8.3)	1 (4.5)	16 (6.8)
BPI-ANCA (MV ± SD) U/mL	76.5 ± 18.8 **	15.6 ± 17.3	(4.10 ± 3.9)
DIV (Propylthiouracil/methimazole), *n* = 17 (%)	0	2 (4.4)	15 (6.8)
BPI-ANCA (MV ± SD) U/mL		13.8 ± 12.9	(2.76 ± 3.2)
AAV, *n* = 40 (%)	1 (4.2)	0	39 (26.5)
BPI-ANCA (MV ± SD) U/mL	77.1 ± 16.5 **		(4.21 ± 3.3)
CTD, *n* = 28 (%)	0	0	28 (19)
BPI-ANCA (MV ± SD) U/mL			(2.19 ± 3.6)
Healthy, *n* = 52 (%)	0	0	52 (35.4)
BPI-ANCA (MV ± SD) U/mL			(3.88 ± 4.1)
All patients, *n* = 193	24	22	147
BPI-ANCA (MV ± SD) U/mL	64.3 ± 17.7 *	32.4 ± 14.1	(3.3 ± 4.1)

BPI-ANCA: Antineutrophil cytoplasmic autoantibodies against bactericidal permeability-increasing protein; UC: Ulcerative colitis; UC/PSC: Ulcerative colitis with primary sclerosing cholangitis; CD: Crohn’s disease; AIH: Autoimmune hepatitis; DIV: Drug-induced vasculitides; AAV: ANCA-associated vasculitides; CTD: Connective tissue diseases. * *p* < 0.05; ** *p* < 0.01; * significance between group of patients with monospecific BPI-ANCA and multispecific BPI-ANCA.

**Table 3:** Specificities and positivity levels for different ANCA antigens in 46 BPI-ANCA-positive patients with prolonged bacterial infections and chronic immunoinflammatory diseases.

ANCA Profile	*Klebsiella*, *Proteus* *mirabilis*, or *Echerichia coli* (*n* = 6)	*Pseudomonas* *Aeruginosa* (*n* = 11)	TBC (*n* = 5)	UC (*n* = 8)	UC/PSC (*n* = 10)	AIH (*n* = 3)	DIV (*n* = 2)	AAV (*n* = 1)
BPI								
Patients, *n* (%)	6 (100)	11 (79)	5 (71)	8 (33)	10 (71)	3 (16)	2 (12)	1 (3)
Low, *n* (%)	3 (50)	1 (10)	3 (43)	5 (21)	5 (36)	2 (11)	2 (12)	1 (3)
Medium, *n* (%)	3 (50)	6 (54)	0	1 (4)	2 (14)	0	0	0
High, *n* (%)	0	4 (36)	2 (29)	2 (8)	3 (21)	1 (5)	0	0
PR3								
Patients, *n* (%)	6 (100)	0	0	3 (13)	8 (57)	1 (5)	2 (12)	0
Low, *n* (%)	3 (50)	0	0	3 (13)	7 (50)	0	1 (6)	0
Medium, *n* (%)	2 (20)	0	0	0	1 (7)	0	1 (6)	0
High, *n* (%)	1 (10)	0	0	0	0	1 (5)	0	0
MPO								
Patients, *n* (%)	3 (50)	0	0	0	0	1 (5)	2 (12)	0
Low, *n* (%)	1 (10)	0	0	0	0	0	1 (6)	0
Medium, *n* (%)	1 (10)	0	0	0	0	0	0	0
High, *n* (%)	1 (10)	0	0	0	0	1 (5)	1 (6)	0
LE								
Patients, *n* (%)	1 (10)	0	0	2 (8)	1 (7)	0	2 (12)	0
Low, *n* (%)	0	0	0	0	0	0	0	0
Medium, *n* (%)	1 (10)	0	0	0	1 (7)	0	1 (6)	0
High, *n* (%)	0	0	0	2 (8)	0	0	1 (6)	0
Cat-G								
Patients, *n* (%)	0	0	0	0	1 (7)	0	2 (12)	0
Low, *n* (%)	0	0	0	0	1 (7)	0	2 (12)	0
Medium, *n* (%)	0	0	0	0	0	0	0	0
High, *n* (%)	0	0	0	0	0	0	0	0
LF								
Patients, *n* (%)	2 (20)	0	0	0	1 (7)	0	1 (6)	0
Low, *n* (%)	1 (10)	0	0	0	1 (7)	0	1 (6)	0
Medium, *n* (%)	0	0	0	0	0	0	0	0
High, *n* (%)	1 (10)	0	0	0	0	0	0	0

TBC: Tuberculosis; UC: Ulcerative colitis; UC/PSC: Ulcerative colitis with primary sclerosing cholangitis; AIH: Autoimmune hepatitis; DIV: Drug-induced vasculitides; AAV: ANCA-associated vasculitides; BPI: Bactericidal/permeability-increasing protein; MPO: Myeloperocsidase; PR3: Proteinase 3; Lf: Lactoferrin; Cat-G: Chatepsin G (CatG); LE: Leukocyte elastase.

**Table 4:** Sensitivity and specificity of BPI-ANCA in 193 patients with persistent infections and immunoinflammatory diseases.

BPI-ANCA Positivity (ELISA)	Sensitivity, *n* (%)	Specificity, *n* (%)
*Pseudomonas aeruginosa*	11/14 (79)	144/179 (80.4)
*Klebsiella pneumoniae*, *Proteus mirabilis* or *Echerichia coli*	6/6 (100)	147/187 (78.6)
*Mycobacterium tuberculosis*	5/7 (71.4)	145/186 (77.9)
UC	8/24 (33.3)	131/169 (77.5)
UC/PSC	10/14 (71.4)	143/179 (79.9)
AIH	3/19 (15.8)	131/174 (75.3)
DIV	2/17 (11.8)	132/176 (75)
AAV	1/40 (2.5)	108/153 (71)
Monospecific BPI-ANCA positivity	Sensitivity, *n* (%)	Specificity, *n* (%)
*Pseudomonas aeruginosa*	11/14 (79)	144/179 (80.4)
*Klebsiella pneumoniae, Proteus mirabilis,* or *Echerichia coli*	0 (0)	163/187 (87.1)
*Mycobacterium tuberculosis*	5/7 (71.4)	145/186 (77.9)
UC	4/24(16.7)	131/169 (77.5)
UC/PSC	1/14 (7.1)	143/179 (79.9)
AIH	2/19 (10.5)	131/174 (75.3)
DIV	0 (0)	152/176 (86.3)
AAV	1/40 (2.5)	108/153 (71)
Multispecific BPI-ANCA positivity	Sensitivity, *n* (%)	Specificity, *n* (%)
*Pseudomonas aeruginosa*	0 (0)	160/182 (87.9)
*Klebsiella pneumoniae, Proteus mirabilis,* or *Echerichia coli*	6/6 (100)	147/187 (78.6)
*Mycobacterium tuberculosis*	0 (0)	164/186 (89.1)
UC	4/24(16.7)	131/169 (77.5)
UC/PSC	9/14 (64.3)	143/179 (79.9)
AIH	1/19 (5.3)	131/174 (75.3)
DIV	2/17 (11.8)	132/176 (75)
AAV	0 (0)	131/153 (85.6)

UC: Ulcerative colitis; UC/PSC: Ulcerative colitis with primary sclerosing cholangitis; AIH: autoimmune hepatitis; DIV: Drug-induced vasculitides; AAV: ANCA-associated vasculitides; BPI: bactericidal/permeability-increasing protein.

## Data Availability

Data are contained within the article. The data presented in this study are available upon request from the corresponding author.

## Abbreviations

ANCA	Antineutrophil cytoplasmic autoantibodies
AAV	ANCA-associated vasculitides
AIH	Autoimmune hepatitis
ASCA	Anti-Saccharomyces cerevisiae antibodies
BPI	Bactericidal/permeability-increasing protein
Cat-G	Catepsin G
CD	Crohn’s disease
CF	Cystic fibrosis
COPD	Chronic obstructive pulmonary disease
CTD	Connective tissue diseases
DIV	Drug-induced vasculitides
ELISA	Enzyme-linked immunosorbent assay
GN	Gram-negative
GP	Gram-positive
GPA	Granulomatosis with polyangiitis
HRP	Horseradish peroxidase
IBD	Inflammatory bowel diseases
IIF	Indirect immunofluorescence
IFN-γ	Interferon γ (IFN-γ)
Il-6	Interleukin 6
Lf	lactoferrin
LAM	Lipoarabinomannan
LE	Leucocyte elastase
LPB	Lipoprotein binding protein
LPS	Lipopolysaccharide
MD-2	Myeloid differentiation factor
MPA	Microscopic polyangiitis
MPO	Myeloperoxidase
OD	Optical densities
OR	Odds Ratio
PR3	Proteinase 3
RPF	Retroperitoneal fibrosis
PSC	Primary sclerosing cholangitis
TBC	Tuberculosis
TMB	Tetramethylbenzidine
TNF-α	Tumor necrosis factor α
UC	Ulcerative colitis
UTI	Urinary tract infection
